# The Neapolitan Phlebotomist

**Published:** 1844-06-29

**Authors:** 


### THE NEAPOLITAN PHLEBOTOMIST.

The taste for blood-letting is universal at Naples. On every the slightest indisposition, or fear of indisposition, all men, women, and children, run to the *Salassatore*, or phlebotomist, to have a little blood drawn from the back of their hand; so that there is not a lad or a young girl of 10 or 12 years of age whose hands do not bear testimony to the repeated applications of the Salassatore's lancet. For a faith which has not a single heretic in the community, of course there is a priesthood—a numerous priesthood. The number of educated medical men would never suffice to perform its offices. This has led to the establishment of a special corporation, whose business it is to handle the lancet, and attach the leech. The phlebotomists have therefore establishments in every street, in every open place at Naples. How often have I paused before the singular insignia by which the shops of these priests of the lancet are distinguished! Imagine to yourself the figure of a man, naked as when he dwelt in paradise, but spirting forth from every vein which steel can reach parabolic jets of blood, an ample pool of which is at the same time collected on the ground. Imagine further, by the side of this awful figure, the effigies of the artist appropriately habited, lancet in hand, and on his knee before his work, like Pygmalion before his statue, and you will have a notion of the way in which the Salassatore here brings the fine arts to his assistance! I was curious to penetrate into one of these sanctuaries of minor surgery, and see its priest close at hand, and seeking some pretext for my intrusion, I demanded a few leeches. I found the phlebotomist at the further extremity of his shop, gravely extended upon a settee of straw, and waiting for a customer with that Neapolitan indifference which resembles at once indolence and sleep, or is in fact a mixture of the two. The shop was poorly furnished, but the walls were occupied from the floor to the roof with a frame-work of little compartments or pigeon holes, filled with compresses and bandages rolled neatly up. I ventured a question on the subject, and learned with amazement that each compartment represented a *customer*, whose fillet and compress were there in readiness. I stepped back a pace, before the sanguinary statistics which the answer of the Neapolitan Salassatore presented to my mind's eye, and did justice at length to the *moderation* of our Parisian phlebotomists who draw blood on the *coup sur coup* system!—*Lon. Med. Gaz., from Gaz. Méd. de Paris.*

